# Relative importance of left atrial reservoir strain compared with components of the HFA-PEFF score: a cross-sectional study

**DOI:** 10.3389/fcvm.2023.1213557

**Published:** 2023-10-12

**Authors:** Minkwan Kim, SungA Bae, Jin Hye Park, In Hyun Jung

**Affiliations:** Division of Cardiology, Department of Internal Medicine, Yongin Severance Hospital, Yonsei University College of Medicine, Yongin-si, Republic of Korea

**Keywords:** left atrial function, heart failure with preserved ejection, reservoir function, left atrial longitudinal strain, HFA-PEFF score

## Abstract

**Background:**

The relative importance of left atrial reservoir strain (LASr) regarding the Heart Failure Association Pre-test assessment, Echocardiography and natriuretic peptide, Functional testing, Final etiology (HFA-PEFF) score, a diagnostic tool for patients with heart failure with preserved ejection fraction (HFpEF), remains unclear. We aimed to identify the relative importance of LASr compared with variables associated with HFpEF and HFA-PEFF scores.

**Methods:**

Between August 2021 and July 2022, we obtained retrospective data from the participants visiting a single cardiovascular center with subjective symptoms of heart failure, such as dyspnea or chest discomfort. In total, 2,712 participants with sinus rhythm and ejection fraction of more than 50% were enrolled. Multivariable logistic regression analysis, random forest analysis, and supervised machine learning algorithms were performed to identify the relative importance of LASr to the HFA-PEFF score.

**Results:**

The average HFA-PEFF score was 2.4 ± 1.6 points. Two hundred and thirty-eight participants had 5 or 6 points. LASr showed a moderate correlation with the HFA-PEFF score (r = −0.50, *p* < 0.001). Impaired LASr < 25.2% was an independent variable affecting a high HFA-PEFF score with traditional diastolic function parameters and components of the HFA-PEFF diagnostic algorithm. The odds ratio (OR) [1.74, 95% confidence interval (CI) 1.23–2.47] for LASr was higher compared to that of left ventricular global longitudinal strain (OR 1.59, 95% CI 1.14–2.21), septal E/e’ (OR 1.23, 95% CI 0.85–1.77), and relative wall thickness (OR 1.20, 95% CI 0.76–1.89). LASr was also a relatively more important variable in estimating a high HFA-PEFF score than TR-Vmax, septal E/e’, septal e’, left ventricular mass index, and relative wall thickness, the major echocardiographic components of the HFA-PEFF score.

**Conclusions:**

LASr is an important factor with components of the HFA-PEFF score and is a useful tool to assess patients with HFpEF.

**Clinical Trial Registration:**

URL: https://clinicaltrials.org. Unique identifiers: NCT05638230.

## 1. Introduction

Heart failure with preserved ejection fraction (HFpEF) accounts for nearly half of all heart failure patients, and as society ages, the incidence of this condition is increasing (1). The causes of HFpEF include dysfunction within the heart, such as cardiovascular disease, and various non-cardiac factors, such as obesity, renal impairment, diabetes mellitus, increased arterial stiffness, systemic inflammation, and frailty (2). Since assessment of diastolic dysfunction is complex and difficult, the 2016 diastolic function guidelines updated by the American Society of Echocardiography and the European Association of Cardiovascular Imaging (ASE/EACVI) stated the possibility of predicting left ventricular diastolic dysfunction using only four echocardiographic parameters (3). Recently, diagnostic algorithms combining echocardiographic parameters, clinical variables, and biomarkers have been proposed for diagnosing HFpEF (4, 5). These algorithms are complex and difficult to apply in real clinical practice; however, as measuring the left ventricular end-diastolic pressure in all patients is not feasible, efforts to validate and utilize these diagnostic algorithms in clinics continue (6).

Left atrial (LA) longitudinal strain, which measures the systolic and diastolic function of the left atrium during the entire cardiac cycle via speckle-tracking echocardiography, has less angle and load dependence and reflects the physiological characteristics according to the cardiac cycle of the left atrium more than conventional echocardiography parameters (7). LA strain (LAS), mainly LA reservoir strain (LASr), is useful for predicting the prognosis and treatment effects in various disease groups, including myocardial disease, heart failure, ventricular tachycardia, and stroke (8–12). In recent studies, LASr also provided additional benefits in diagnosing HFpEF (13, 14). However, there is no study regarding the relative importance and correlation of LASr with traditional cardiovascular risk factors and established echocardiographic parameters. We aimed to understand and utilize LASr more effectively in clinical practice by determining its relative importance compared to components of HFpEF diagnostic algorithms that are not yet used in guidelines.

## 2. Materials and methods

### 2.1. Study participants

**Figure 1:**
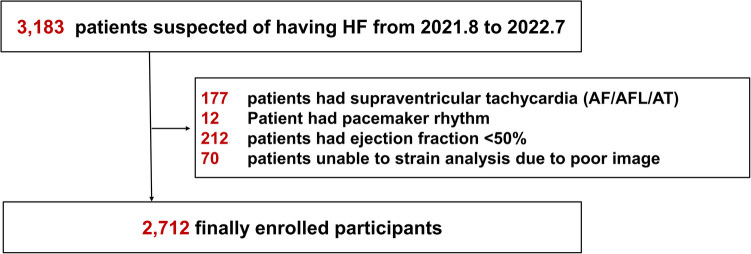
Flowchart of the study. HF, heart failure; AF, atrial fibrillation; AFL, atrial flutter; AT, atrial tachycardia.

Between August 2021 and July 2022, we retrospectively analyzed data from 3,183 patients aged ≥20 years who visited our cardiovascular center with subjective symptoms of heart failure, such as dyspnea or chest discomfort. Participants underwent transthoracic echocardiography (TTE) and had an International Classification of Disease-10 code of 150 (heart failure) (URL: https://clinicaltrials.org. Unique identifiers: NCT05638230). Patients were excluded based on the following parameters: supraventricular arrhythmia such as atrial fibrillation/flutter or atrial tachycardia (*n* = 177); left ventricular ejection fraction < 50% (*n* = 212); permanent pacemaker or implantable cardioverter defibrillator procedure (*n* = 12); and unable to complete strain analysis because of poor imaging (*n* = 70). In total, 2,712 participants were enrolled (Figure 1). This study was conducted based on the revised Helsinki Declaration of 2013 and approved by the Institutional Review Board of our hospital (IRB number: 9-2022-0101). The requirement for informed consent was waived because it was a retrospective study.

### 2.2. Data collection

We obtained participant information using the clinical data server analysis system, Severance Clinical Research Analysis Portal. Medical history, including hypertension, diabetes mellitus, chronic kidney disease, previous coronary revascularization, and previous stroke, was also obtained using the same analysis system. The operational definitions are summarized in Supplementary Table S1. As laboratory findings, we collected hemoglobin, glucose, glycated hemoglobin, creatinine, NT-proBNP, and total cholesterol levels and calculated the glomerular filtration rate using the Chronic Kidney Disease Epidemiology Collaboration equation (15). The TTE examination performed on participants using the commercially available echocardiographic vendor (Vivid E9/E95, GE HealthCare, Horton, Norway). The traditional echocardiographic parameters and left ventricular global longitudinal strain (LVGLS) were collected based on the guidelines (3, 16). The ejection fraction of the left ventricle (LV) was evaluated using Simpson's biplane method.

### 2.3. LAS analysis

LAS was measured using commercially available software (EchoPAC version 204, AFI LA 3.0, GE HealthCare, Horton, Norway) through speckle-tracking and semiautomatic analysis methods per the guidelines (17). An experienced sonographer blinded to clinical information measured LAS. LAS was calculated from the non-foreshortened apical four-chamber view. The start point of the R-wave was used as the time reference; when this was not clear, the nadir of the LAS curve was defined as end-diastole (17). The region of interest (ROI) was automatically traced by selecting the septal and lateral parts of the proximal portion of the mitral annulus and the LA roof and adjusted to match the endocardial border of the LA as needed. The ROI was measured considering anatomical LA wall thickness by setting it as thin as possible (maximal thickness of 3 mm) to avoid including the pericardium. Care was taken to measure it without including the pulmonary vein and LA appendage (Supplementary Figure S1). The LAS curve was measured by dividing it into three phases: LASr, which is the peak value from the nadir of the LA strain curve; LA conduit strain (LAScd), calculated by subtracting the value at the time of start of atrial contraction from the value at mitral valve opening; and LA contraction strain (LASct), the difference between the end-diastolic strain of the ventricle and the value at the start of atrial contraction. We used the LAS obtained from the non-foreshortened apical four-chamber view. All LAS values are presented as absolute values throughout the manuscript for comparison convenience.

### 2.4. Study endpoint

The endpoint was the relative importance of the LAS in predicting a high Heart Failure Association Pre-test assessment, Echocardiography and natriuretic peptide, Functional testing, Final etiology (HFA-PEFF) diagnostic algorithm score of 5 or 6 compared to components of the 2016 guideline for diastolic function and the HFA-PEFF diagnostic algorithm.

### 2.5. Statistical analysis

The values of each variable were presented as mean ± standard deviation or median (interquartile range) based on the fulfillment of normality for continuous variables and as numbers and percentages for categorical variables. The continuous variables were compared using Student's *t*-test, while the categorical variables were compared using the chi-squared test. Missing data were evaluated using the MssForest algorithm (18). The best cutoff value of LASr for predicting a high HFA-PEFF score was calculated using the Youden index (19). We used multivariable binary logistic regression to identify the factors predicting a high HFA-PEFF score by comparing it to the components of the ASE/EACVI 2016 diastolic function evaluation guideline and the HFA-PEFF diagnostic algorithm. Random forest analysis was used to evaluate the relative importance of LASr in contributing to a high HFA-PEFF score among demographic, clinical, and laboratory covariates. The group was divided into derivation (65%) and validation (35%) cohorts. This was done to evaluate the performance of the optimized model created from the derivation cohort using the receiver operating characteristic analysis, which was measured by the area under the curve. To determine the significance of each variable, the random forest trees were analyzed based on classification error, and the impact of each predictor variable was assessed by permuting it and measuring the resulting error. To observe changes in diagnostic performance upon substituting LASr for some variables in the existing models, we performed analyses of the net reclassification index (NRI) and integrated discrimination improvement (IDI). Two-tailed *p* < 0.05 was considered statistically significant. All statistical analyses were performed using R version 4.1.2 software (R Development Core Team, Vienna, Austria).

## 3. Results

### 3.1. Baseline characteristics and echocardiographic parameters, including LAS

The baseline characteristics of 2,712 participants are described in Table 1. The mean age was 62.1 ± 16.5, and 53.0% were women. Participants tended to be obese (average body mass index: 25.0 ± 4.0 kg/m^2^), half of them had a history of hypertension, and 28.0% had a history of diabetes mellitus, which are risk factors for HFpEF (Table 1). The prevalence rates of chronic kidney disease, coronary artery disease needing revascularization, and previous stroke were 11.7%, 7.7%, and 7.2%, respectively. The mean NT-proBNP was higher than the reference value of 125 pg/ml (953.2 ± 4779.4 pg/ml).

**Table 1:** Clinical characteristics of study participants.

Characteristic	Value (*n* = 2,712)
Age, years	62.1 ± 16.5
Female sex	1,437 (53.0)
Weight, kg	65.8 ± 13.7
Height, cm	162.0 ± 9.9
Body mass index, kg/m^2^	25.0 ± 4.0
Systolic blood pressure, mmHg	133.1 ± 18.4
Diastolic blood pressure, mmHg	74.8 ± 13.7
Heart rate, beats per minute	69.2 ± 12.3
Cardiovascular risk factors	
Hypertension	1,366 (50.4)
Diabetes mellitus	759 (28.0)
Chronic kidney disease	318 (11.7)
Previous coronary revascularization	208 (7.7)
Previous stroke	194 (7.2)
Laboratory findings	
Hemoglobin, g/dl	13.2 (12.0–14.5)
Random glucose, mg/dl	104.0 (95.0–121.0)
Glycated hemoglobin, %	5.9 (5.6–6.3)
Estimated glomerular filtration rate, ml/min/1.73 m^2^	91.7 (78.8–102.2)
NT-proBNP, pg/ml	131.6 (62.0–347.0)
Total cholesterol, mg/dl	162.0 (132.0–191.0)

Numbers are presented as mean ± SD, median (interquartile range) as appropriate, or *n* (%).

The results of traditional echocardiographic and strain parameters are presented in Table 2. The mean value of the LV ejection fraction was 62.6 ± 4.8%. Over half of the participants (66.4%) exhibited decreased septal e’ velocity < 7 cm/s. Three hundred twenty-three participants (11.9%) had a septal E/e’ ≥15, and 197 participants (7.3%) presented with mitral annular calcification. The proportion of participants with septal E/e’ ≥15 was significantly higher among those with mitral annular calcification than those without (39.1% vs. 9.8%, *p* < 0.001) (Supplementary Table S2). The mean LA volume index was 31.5 ± 11.2 ml/m^2^. The mean LVGLS, LASr, LAScd, and LASct were 17.4 ± 2.5, 28.9 ± 8.9, 15.7 ± 7.7, and 13.4 ± 5.5%, respectively.

**Table 2:** Results of echocardiographic variables.

	Value (*n* = 2,712)
LV end-diastolic dimension, mm	47.0 ± 4.4
LV end-systolic dimension, mm	30.8 ± 3.7
LV ejection fraction, %	62.6 ± 4.8
Relative wall thickness	0.36 ± 0.60
LV mass index, g/m^2^	79.6 ± 34.5
Septal e’ velocity, cm/s	6.5 ± 2.4
Septal e’ velocity < 7 cm/s	1,789 (66.4)
Septal E/e’	10.7 ± 4.2
Septal E/e’ ≥ 15	323 (11.9)
Mitral annular calcification	197 (7.3)
LAVI, ml/m^2^	31.5 ± 11.2
LAVI ≥ 34 ml/m^2^	949 (35.0)
TR-Vmax, m/s	2.4 ± 0.3
TR-Vmax > 2.8 m/s	267 (9.8)
LV global longitudinal strain, %	17.4 ± 2.5
LA reservoir strain, %	28.9 ± 8.9
LA conduit strain, %	15.7 ± 7.7
LA booster pump strain, %	13.4 ± 5.5

Numbers are presented as mean ± SD or *n* (%).

### 3.2. Association between LAS and diastolic function parameters

**Figure 2:**
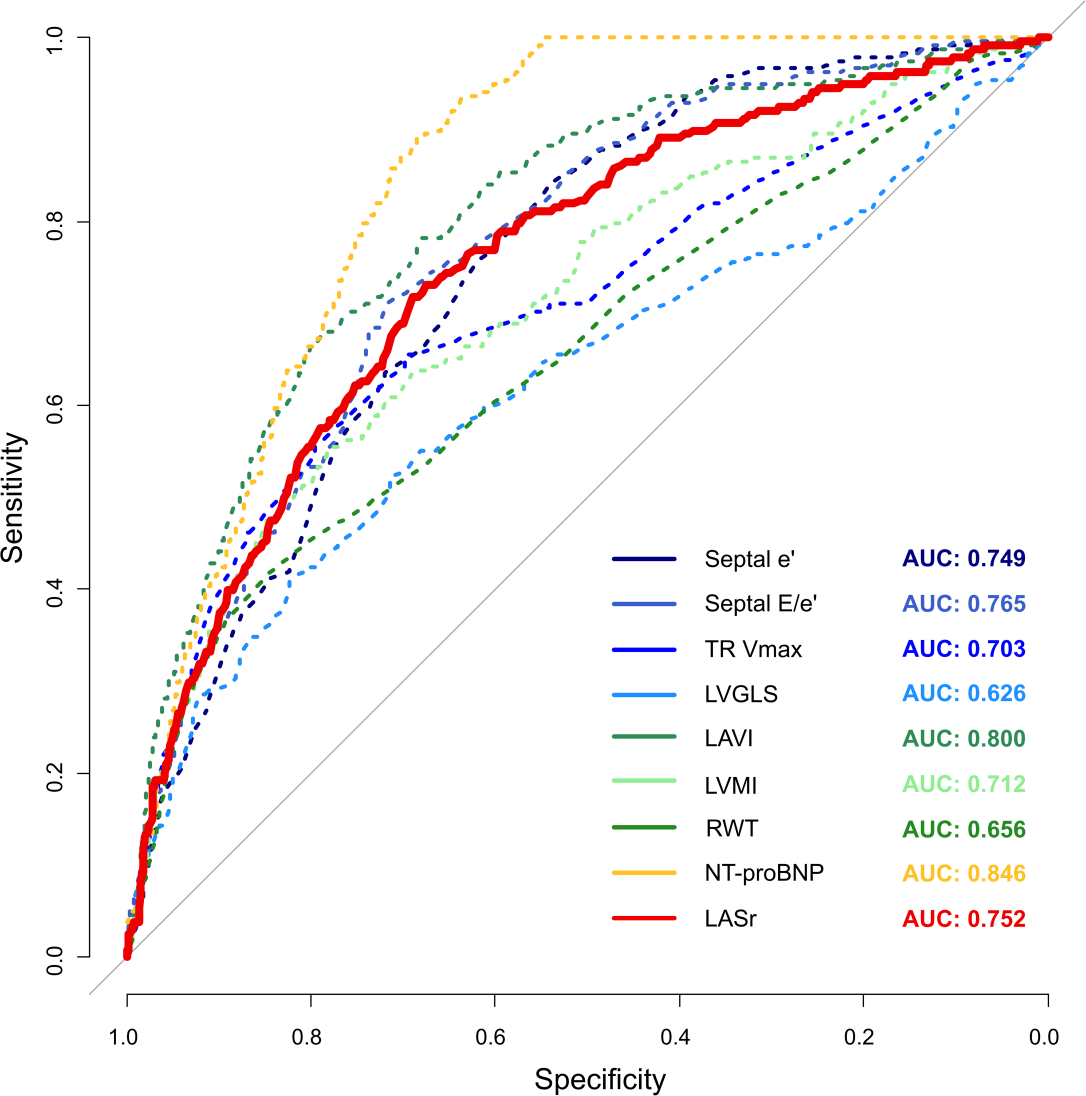
Diagnostic performance of LASr to estimate a high HFA-PEFF score with other components consisting of the HFA-PEFF diagnostic algorithm.

The average HFA-PEFF score in the total population was 2.4 ± 1.6 points, and 238 (8.8%) had a high HFA-PEFF score. LASr, previously identified as a useful LAS parameter, was moderately correlated with the HFA-PEFF score (r = −0.50, *p* < 0.001). In the receiver operating characteristic curve analysis, LASr had good diagnostic performance to estimate the HFA-PEFF score [sensitivity 71.8%, specificity 68.9%, area under the curve 0.75, 95% confidence interval (CI) 0.72–0.78]. The area under the curve of LASr was similar to or higher than that of traditional diastolic function parameters [0.77 (0.74–0.79) in septal E/e’, 0.75 (0.72–0.78) in septal e’, 0.70 (0.66–0.74) in maximal velocity of tricuspid regurgitation (TR-Vmax)] (Figure 2).

Among diagnostic function parameters, there were moderate correlations between LASr and three of four major diastolic function parameters [r = 0.55 in septal e’, r = −0.43 in septal E/e’, r = −0.41 in LA volume index (LAVI); all *p*’s < 0.001] and LVGLS (r = 0.37, *p* < 0.001). LASr was found to be mildly correlated with the peak velocity of tricuspid regurgitation (r = −0.25, *p* < 0.001) and relative wall thickness (RWT; r = −0.26, *p* < 0.001) and had a very weak correlation with NT-proBNP (r = −0.12. *p* < 0.001). In addition, LASr was found to be was moderately correlated with the number of abnormal diastolic function parameters from the ASE/EACVI 2016 diastolic function guideline (r = −0.52, *p* < 0.001).

### 3.3. Relative importance of LASr with components of the ASE/EACVI 2016 diastolic function guideline and the HFA-PEFF diagnostic algorithm

We performed a multivariable logistic regression analysis of two different models to identify the relative importance of LASr with components of the ASE/EACVI 2016 diastolic function guideline and the HFA-PEFF diagnostic algorithm (Table 3). In model 1 (2016 ASE/EACVI diastolic function guideline and LASr), LASr was an independent parameter to estimate a high HFA-PEFF score with other diastolic function parameters [odds ratio (OR) 2.39, 95% CI 1.72–3.30, *p* < 0.001]. The OR of LARs < 25.2% was higher than that of septal E/e’ (OR 1.41, 95% CI 1.00–1.99, *p* = 0.049), which is a useful parameter to estimate LV filling pressure in real clinical practice. In model 2 (HFA-PEFF diagnostic algorithm and LASr), impaired LASr < 25.2% also had a significant role in predicting a high HFA-PEFF score with covariates (OR 1.74, 95% CI 1.23–2.47; *p* = 0.002) (Table 3). LASr < 25.5% had a higher OR than septal E/e’ (OR 1.41), LVGLS < 16% (OR 1.59), and RWT (OR 1.20).

**Table 3:** Multivariable logistic regression models showing the association between a high HFA-PEFF score of 5 or 6 and the LA reservoir strain with parameters consisting of the 2016 ASE/EACVI diastolic function guideline and the HFA-PEFF score.

Dependent variable	OR (95% CI)	*p* value
Model 1 (ASE/EACVI diastolic function guideline + LASr)		
Septal E/e’ > 15	1.41 (1.00–1.99)	0.048
Septal e’ < 7 cm/s	5.46 (2.90–10.29)	<0.001
LAVI > 34 ml/m^2^	3.32 (2.37–4.67)	<0.001
TR-Vmax > 2.8 m/s	2.80 (2.00–3.92)	<0.001
LASr < 25.2%	2.38 (1.72–3.30)	<0.001
Model 2 (HFA-PEFF diagnostic algorithm + LASr)		
Septal E/e’ > 15	1.23 (0.85–1.78)	0.264
Septal e’ < 7 cm/s	4.01 (2.10–7.66)	<0.001
LAVI ≥ 34 ml/m^2^	3.22 (2.25–4.62)	<0.001
TR-Vmax > 2.8 m/s	2.81 (1.96–4.03)	<0.001
LVMI > 115 g/m^2^ for men or >95 g/m^2^ for women	3.83 (2.58–5.69)	<0.001
RWT > 0.42	1.21 (0.77–1.90)	0.420
NT-proBNP > 125 pg/ml	2.43 (1.50–3.94)	<0.001
LVGLS < 16%	1.59 (1.14–2.22)	0.006
LASr < 25.2%	1.74 (1.23–2.47)	0.002

**Figure 3:**
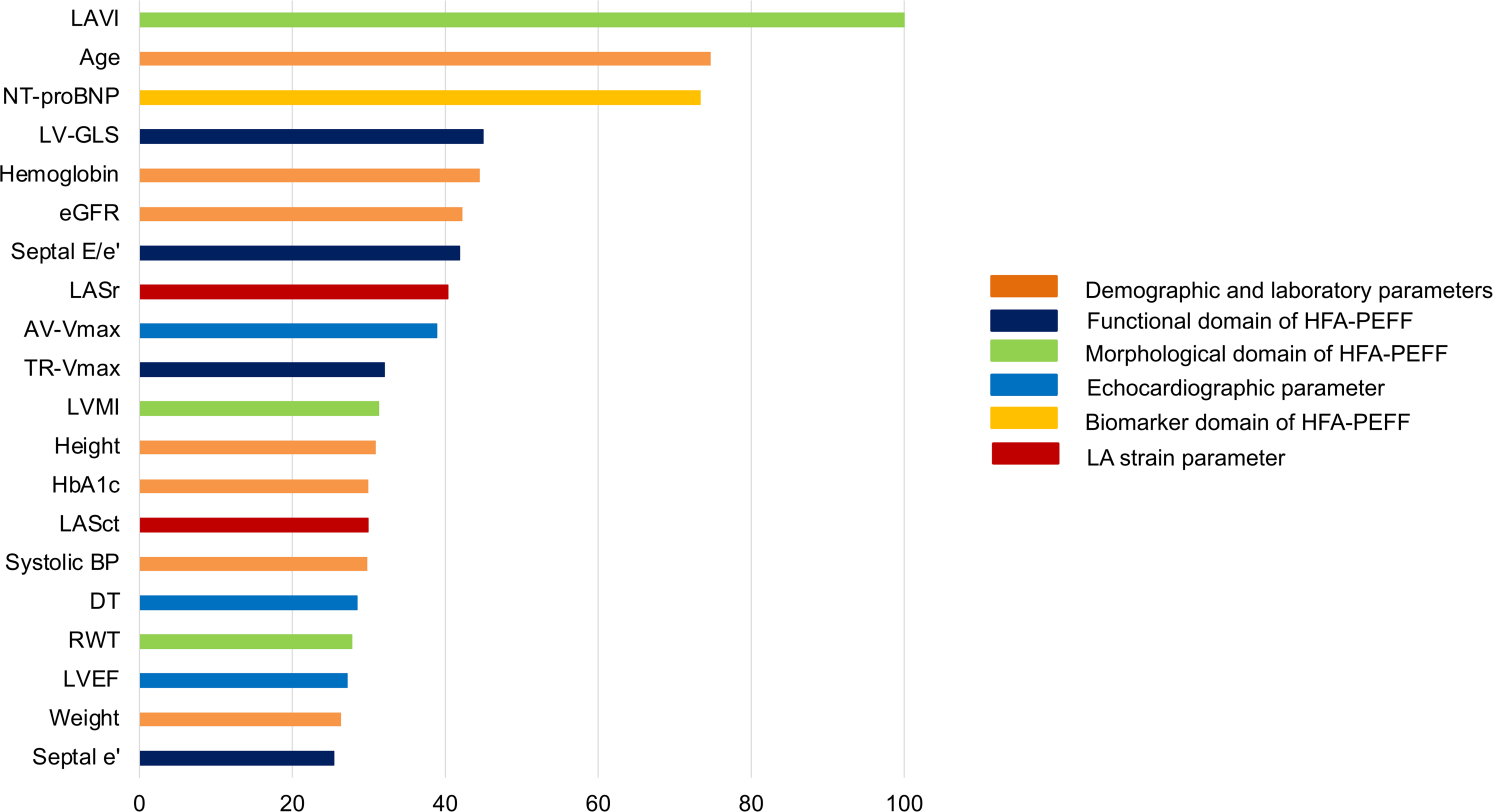
Relative importance of LAS in predicting a high HFA-PEFF score of 5 or 6 as analyzed in random forest analysis. Among 36 variables, only the top 20 parameters are shown. The highest important variable (LAVI) was set as 100, and other variables were compared to identify the relative importance.

We conducted random forest analysis to analyze the relative importance of LAS (LASr, LAScd, and LASct) in predicting a high HFA-PEFF score in relation to 33 other demographic, clinical, laboratory, and echocardiographic covariates. The best-predicting model generated from the training cohort was built on a model of 1,500 tree with an area under the curve of 0.91 (95% CI 0.89–0.92), as evaluated on the validation cohort. Along with LAVI, NT-proBNP, age, LVGLS, hemoglobin, and glomerular filtration rate, LASr had a higher importance in predicting a high HFA-PEFF score than traditional diastolic parameters such as E/e’, TR-Vmax, LV mass index (LVMI), RWT, and septal e’ (Figure 3 and Supplementary Table S3). Presuming the significance of the highest variable (LAVI) to be 100, the relative importance of LASr in a high HFA-PEFF score was 40.3, which was similar to or higher than that of traditional diastolic function parameters (41.9 in septal E/e’, 32.0 in TR-Vmax, 31.3 in LVMI, 27.8 in RWT, and 24.7 in septal e’).

### 3.4. Changes in the diagnostic performance for predicting a high HFA-PEFF score upon substituting LASr for variables in existing models

**Figure 4:**
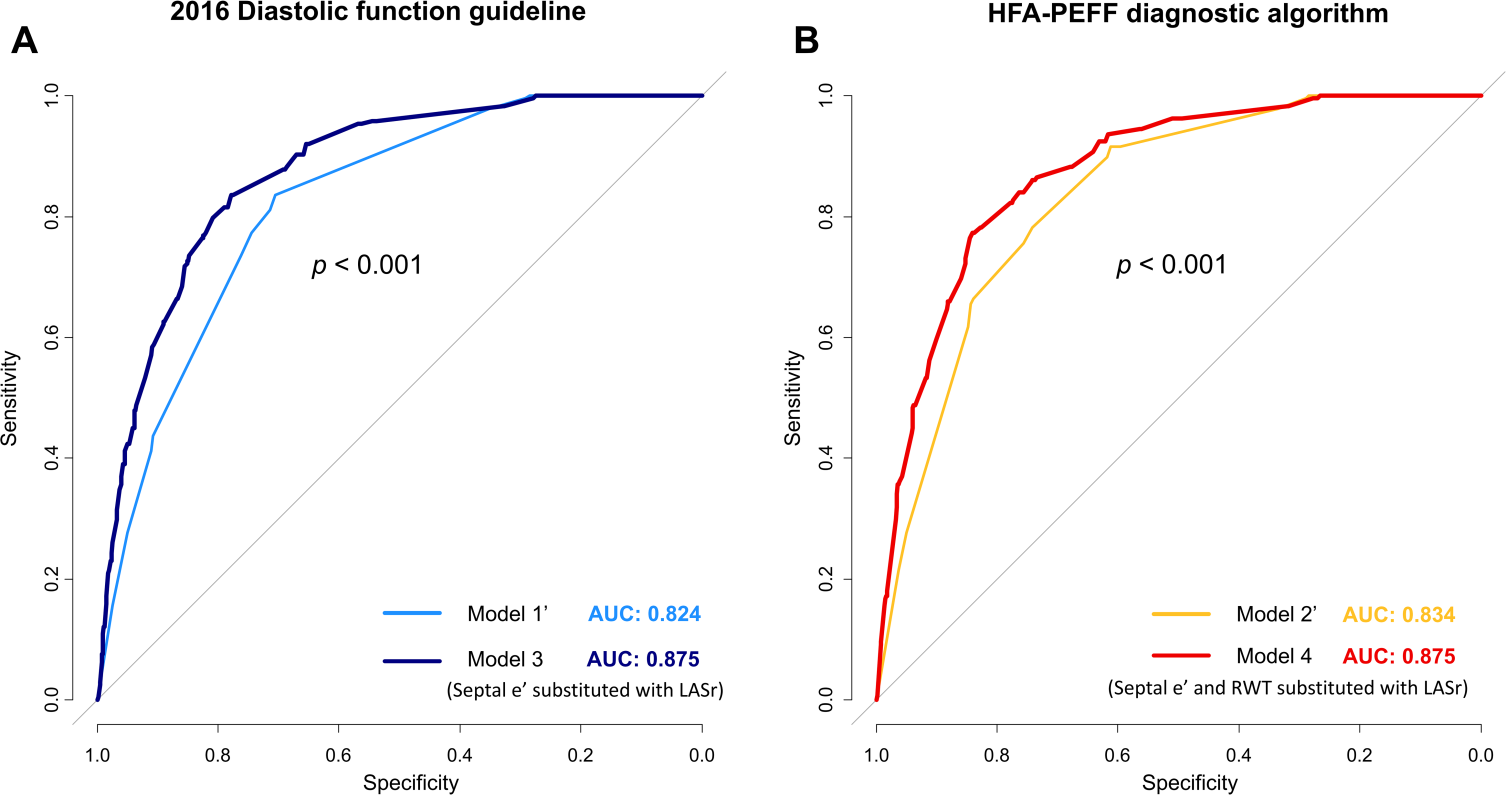
Changes in diagnostic performance for predicting a high HFA-PEFF score upon substituting LASr for variables in the 2016 diagnostic function guideline (**A**) and HFA-PEFF diagnostic algorithm (**B**).

Incorporating LASr into existing models for predicting diastolic dysfunction and HFpEF removed variables with lower ORs or statistical insignificance and demonstrated the enhanced diagnostic performance of these revised models to estimate high HFA-PEFF scores (Figure 4 and Table 4). In the newly constructed model (model 3) where LASr replaced septal E/e’, which had a lower OR value in the previous 2016 diastolic function guideline model (model 1’), the performance for predicting a high HFA-PEFF score improved—an increase in the C-statistics value from 0.824 to 0.875, and the NRI and IDI of the new model exhibited enhanced diagnostic performance with values of 0.937 (95% CI 0.814–1.061) and 0.081 (95% CI 0.063–0.099), respectively (Table 4 and Figure 4A). Another newly formulated model (model 4), which incorporated LASr while excluding septal E/e’ and RWT—variables that had lower OR values than LASr in the original HFA-PEFF model (model 2’)—demonstrated statistically significant superior performance compared to the traditional HFA-PEFF algorithm [C-statistics 0.834–0.875; *p* < 0.001, NRI 0.910 (95% CI 0.784–1.035), IDI (0.074, 95% CI 0.058–0.091)] (Table 4 and Figure 4B).

**Table 4:** Comparison of diagnostic performance of models predicting an HFA-PEFF score of 5 or 6 points.

	C-statistics		Net reclassification index		Integrated discrimination improvement	
	95% CI	*p* value	95% CI	*p* value	95% CI	*p* value
Model 1’ (2016 diastolic function guideline)	0.824 (0.801–0.847)					
Model 3 (Model 1’—septal E/e’ + LASr)	0.875 (0.855–0.896)	<0.001*	0.937 (0.814–1.061)	<0.001	0.081 (0.063–0.099	<0.001
Model 2’ (HFA-PEFF algorithm)	0.834 (0.812–0.857)					
Model 4 [Model 2’—(septal E/e’ and RWT) + LASr]	0.875 (0.854–0.895)	<0.001^†^	0.910 (0.784–1.035)	<0.001	0.074 (0.058–0.091)	<0.001

Model 1’ consists of components based on the 2016 diastolic function guideline: septal e’, septal E/e’, TR-Vmax, and LAVI.

Model 2’ consists of components based on HFA-PEFF diagnostic algorithms: septal e’, septal E/e’, TR-Vmax, LAVI, RWT, left ventricular mass index, left ventricular global longitudinal strain, and NT-proBNP.

* Compared with Model 1’.

^†^ Compared with Model 2’.

## 4. Discussion

The present study showed that LAS, especially LASr, was independently associated with a high HFA-PEFF score and good performance to diagnose HFpEF and was similar or a more important parameter than traditional diastolic function parameters such as septal E/e’. In contrast to previous studies that focused on LASr as an independent or incremental predictor for prognosis in patients with heart failure, this study aimed to examine the relative importance of LAS compared to existing parameters for assessing diastolic function. Although LAS is not yet used in the diagnosis algorithm or guidelines for HFpEF, demonstrating the usefulness of LAS could serve as evidence for its potential utilization in the diagnosis of HFpEF.

LAS is a useful indicator to predict a poor cardiovascular prognosis, including readmission, in patients with heart failure (HF) (12, 20). A previous study suggested that LASr is the most important predictor of poor cardiovascular outcomes among cardiac mechanics indicators in patients with HFpEF (21). LASr has also proven helpful in predicting incident HF in individuals with asymptomatic risk factors (22). Moreover, replacing LAVI with LASr more effectively reclassified indeterminate decisions to normal in all cases of diastolic function analysis (22). Conversely, impaired LAS has been reported to predict worse NYHA functional class and elevated estimated right ventricular systolic pressure, even in patients with normal LAVI (23). As LASr progressively changes according to the severity of diastolic dysfunction, it effectively categorizes diastolic dysfunction grading in the existing ASE/EACVI 2016 algorithm (24). In our study, LASr demonstrated similar importance to existing parameters in predicting the possibility of HFpEF; therefore, it may be used in HFpEF diagnosis in the future. In addition to LASr, LAScd and LASct are highly associated with tissue Doppler parameters and represent indicators of LA afterload (LV end-diastolic pressure) and LA pumping function (25, 26). Our results in random forest analysis also demonstrated that LASct had comparable importance to previous important parameters, thus helping to explain the clinical utility of LAS.

The HFA-PEFF diagnostic algorithm was developed to diagnose HFpEF more accurately, which was previously difficult and challenging (4). The algorithm confirms the likelihood of HFpEF through a pre-test assessment of the ambulatory setting, followed by score calculation using TTE and NT-proBNP. The algorithm was validated in two independent prospective cohorts and was demonstrated to be helpful in diagnosing HFpEF (6). Unlike the H2FPEF, another algorithm to diagnose HFpEF, the HFA-PEFF includes a wider range of diastolic function-associated echocardiographic parameters, which increases accuracy while retaining the ASE/EACVI 2016 algorithm (3, 4). However, when tested on a large cohort of patients presenting with dyspnea, the HFA-PEFF diagnostic algorithm was unable to exclude a significant number of healthy participants and diagnosed a group of patients that did not overlap with those diagnosed based on the ASE/EACVI 2016 and H2FPEF algorithms (27). Our study demonstrated the importance of LAS that was comparable to the parameters required for the HFA-PEFF in HFpEF diagnoses. Therefore, LAS can be used as an additional auxiliary or substitute tool in HFpEF diagnosis.

The E/e’ is a long-standing predictor of LV filling pressure and is an important indicator for evaluating diastolic function. However, conditions such as annular calcification, mitral regurgitation, and pericardial disease may not accurately reflect LV filling pressure (3). Among the participants of our study, those with mitral annular calcification also tended to have higher septal E/e’; thus, the predictive power of E/e’ to estimate a high HFA-PEFF score may have been lower than that of LASr. The LASr cutoff value in our study predicted a high HFA-PEFF score of 25.2, which was higher than the previously proposed score of 18 (9); however, this was similar to the average value of 24.6 in the PARAMOUNT trial and the average of the apical four- and two-chamber strain value of 26.0 in the TOPCAT trial (12, 28). There are two reasons to explain this discordance. First, differences in the software used to measure LAS may account for the variation. However, a recent study showed no significant difference in strain values depending on the software used for analysis (29). Second, we tried to trace only the thin LA wall to avoid the pericardial tissue and set the ROI as light as possible by setting the ROI to the default value of 3 mm or less while ensuring that tracing was possible (Supplementary Figure S1) (17). Among speckle-tracking echocardiographic strain analysis software, TOMTEC tends to show higher strain values than EchoPAC (30). However, LAS values would be similar to those measured by TOMTEC if the ROIs were set as thin as possible to trace. Moreover, the main focus of our study was not the LASr cutoff value but rather the potential of LAS to provide additional assistance in diagnosing diastolic dysfunction and HFpEF.

AF patients, who were excluded from our study, show a significant decrease in LASr compared to that in patients in sinus rhythm, a phenomenon that can be explained mechanistically by the absence of booster pump action and the atrioventricular dyssynchrony. In previous studies, LASr in patients with AF was lower than those in the healthy population, with LASr values averaging approximately 10%–15% (31, 32). Another study observed a decrease in LASr even in AF patients with normal invasively measured LV filling pressure (9). This potential decrease in LASr in patients with AF in our study cohort may confound the main purpose of our study, which was to help in the differential diagnosis of HFpEF patients with sinus rhythm. Previous studies also consistently excluded patients with AF, other supraventricular arrhythmias, and pacemaker rhythms from analysis of LA strain (11, 33).

Our study had some limitations. First, the study was designed retrospectively; thus, the power of the evidence may be weaker than that of a prospective or randomized control study. However, we tried to overcome any shortcomings of a retrospective study by measuring LAS across a large cohort. Furthermore, various models and statistical methods were used to demonstrate the concordant significance of LAS. Second, the primary endpoint of our study was not an indicator measured through invasive measurement or a hard endpoint, such as cardiovascular mortality. However, the HFA-PEFF score is an excellent and validated diagnostic algorithm. In addition, for scores of five or higher, the sensitivity and positive predictive value for diagnosing HFpEF were very high, at 93% and 98%, respectively (6). Third, interactions between LAS and other diastolic functions and echocardiographic parameters are possible. Recent studies have suggested that LAS is closely associated with not only LV function but also various indices such as e’ and a’ (9, 25, 26). However, LAS can be used in clinical practice as a reproducible, single index that can integrate various echocardiographic indices and produce an easily understood numerical value. Based on our results, well-designed future studies are needed to accumulate evidence to include LAS in the guidelines for diagnosing HFpEF.

## 5. Conclusion

LASr is a relatively important factor with components of the HFA-PEFF score, a useful tool to assess patients with HFpEF. Ultimately, additional efforts are needed to incorporate LAS into the diagnostic process for HFpEF.

## Data Availability

The original contributions presented in the study are included in the article/**Supplementary Material**, further inquiries can be directed to the corresponding author.
