# Supplementary material for: Relative importance of left atrial reservoir strain compared with components of the HFA-PEFF score: a cross-sectional study
Source: Front Cardiovasc Med. 2023 Oct 12;10:1213557. doi: 10.3389/fcvm.2023.1213557 (PMC10602785; doi:10.3389/fcvm.2023.1213557)
Supplement: Supplementary file 1 [file Datasheet1.docx]

**Supplementary Tables**

**Table S1. Operational definitions of covariates**

| **Diagnosis** | **ICD-10-CM code and definition** | **Diagnostic definition** |
| --- | --- | --- |
| **Hypertension** | I10-I13, I15; and minimum 1 prescription of anti-hypertensive drug (thiazide, loop diuretics, aldosterone antagonist, alpha-/beta-blocker, calcium channel blocker, renin-angiotensin system blocker). | Admission ≥1 or outpatient department ≥2 |
| **Diabetes mellitus** | E11-E14; and minimum 1 prescription of anti-diabetic drugs (sulfonylureas, metformin, meglitinides, thiazolidinediones, dipeptidyl peptidase-4 inhibitors, α-glucosidase inhibitors, and SGLT2-inhibitor, GLP-1 agonist, or insulin). | Admission ≥1 or outpatient department ≥2 |
| **Chronic kidney disease** | N00-007, N11, I12, N18-19, Q61 | Estimated glomerular filtration rate < 60 mL/min/1.73 m^2^ by Chronic Kidney Disease Epidemiology Collaboration (CKD-EPI) equation |
| **Dyslipidaemia** | E78, and minimum 1 prescription of lipid-lowering medication (statin, ezetimibe, fenofibrate) | Admission ≥1 or outpatient department ≥2 |
| **Coronary revascularization** | I20, I23-25, and have the procedure code of M6561-6567 | Admission ≥ 1 or outpatient clinic ≥ 2 |
| **Previous stroke** | I63, I64; and brain imaging either brain magnetic resonance or computed tomography within 7 days | Admission ≥1 or outpatient clinic ≥ 2 |
|  |  |  |

**Table S2. The proportion of participants with septal E/e' ≥15 based on the presence of mitral annular calcification**

|  | MAC (+)  (N=197) | MAC (-)  (N=2,515) | *P* value |
| --- | --- | --- | --- |
| Septal E/e’ ≥15 | 77 (39.1) | 246 (9.8) | <0.001 |
| Septal E/e’ <15 | 120 (60.9) | 2,269 (90.2) |  |

Numbers are presented as mean ± SD or n (%). MAC, mitral annular calcification

**Table S3. Random forest analysis results for relative variable importance in predicting high HFA-PEFF score of 5 or 6**

| **Variables** | **Overall** | **Variables** | **Overall** |
| --- | --- | --- | --- |
| LAVI | 100.0 | LVMI | 31.3 |
| Age | 74.7 | Height | 30.9 |
| NT-proBNP | 73.3 | HbA1c | 29.9 |
| LV-GLS | 44.9 | LASct | 29.9 |
| Hemoglobin | 44.5 | Systolic BP | 29.8 |
| eGFR | 42.2 | DT | 28.5 |
| E/e' (septal) | 41.9 | RWT | 27.8 |
| LASr | 40.3 | LVEF | 27.2 |
| AV-Vmax | 38.9 | Weight | 26.4 |
| TR-Vmax | 32.0 | Septal s' | 25.4 |

Among 36 variables, only the 20 top important variables are shown in the table. LAVI, left atrial volume index; LV-GLS, left ventricular global longitudinal strain; GFR, glomerular filtration rate; LASr, left atrial reservoir strain; AV-Vmax, transaortic peak velocity; TR-Vmax, peak tricuspid regurgitation velocity; LASct, left atrial contraction strain; LVMI, left ventricular mass index; BP, blood pressure; DT, deceleration time; LVEF, left ventricular ejection fraction; RWT, relative wall thickness.

**Supplementary Figures**

**
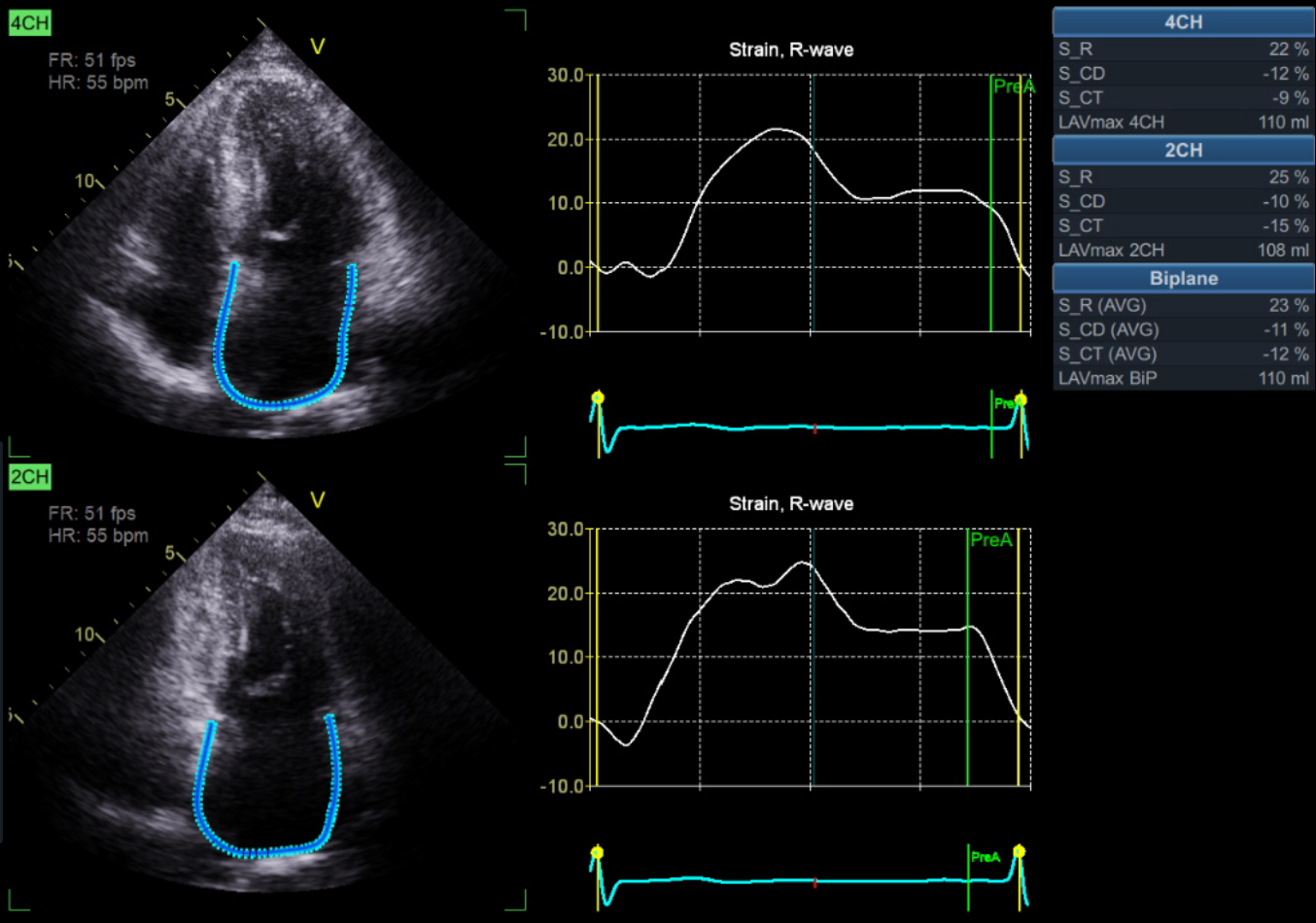
**

**Figure S1. Representative example to measure LAS.**
